# Acute-On-Chronic Liver Failure Defined by Asian Pacific Association for the Study of the Liver Should Include Decompensated Cirrhosis

**DOI:** 10.3389/fmed.2021.750061

**Published:** 2021-10-15

**Authors:** Manman Xu, Ming Kong, Pengfei Yu, Yingying Cao, Fang Liu, Bing Zhu, Yizhi Zhang, Wang Lu, Huaibin Zou, Shaoli You, Shaojie Xin, Zhongping Duan, Tao Han, Yu Chen

**Affiliations:** ^1^Fourth Department of Liver Disease (Difficult & Complicated Liver Diseases and Artificial Liver Center), Beijing You'an Hospital Affiliated to Capital Medical University, Beijing, China; ^2^Beijing Municipal Key Laboratory of Liver Failure and Artificial Liver Treatment Research, Beijing, China; ^3^Department of Hepatology, Third Central Hospital of Tianjin, Tianjin, China; ^4^Liver Failure Treatment and Research Center, The Fifth Medical Center of Chinese People's Liberation Army (PLA) General Hospital, Beijing, China

**Keywords:** Asian Pacific Association for the Study of the Liver (APASL), acute-on-chronic liver failure (ACLF), diagnostic indicator, mortality, decompensated cirrhosis

## Abstract

**Background and Aims:** Acute-on-chronic liver failure (ACLF) is an acute deterioration of chronic liver disease with high short-term mortality. The inclusion or exclusion of previously decompensated cirrhosis (DC) in the diagnostic criteria of ACLF defined by the Asian Pacific Association for the Study of the Liver (APASL-ACLF) has not been conclusive. We aimed to evaluate the prognostic impact of decompensated cirrhosis in ACLF.

**Methods:** We retrospectively collected a cohort of patients with a diagnosis of APASL-ACLF (with or without DC) hospitalized from 2012 to 2020 at three liver units in tertiary hospitals. Baseline characteristics and survival data at 28, 90, 180, 360, 540, and 720 days were collected.

**Results:** Of the patients assessed using APASL-ACLF criteria without the diagnostic indicator of chronic liver disease, 689 patients were diagnosed with ACLF, of whom 435 had no decompensated cirrhosis (non-DC-ACLF) and 254 had previously decompensated cirrhosis (DC-ACLF). The 28-, 90-, 180-, 360-, 540-, and 720-day mortality were 24.8, 42.9, 48.7, 57.3, 63.4, and 68.1%, respectively, in DC-ACLF patients, which were significantly higher than in non-DC-ACLF patients (*p* < 0.05). DC was independently associated with long-term (180/360/540/720 days) but not short-term (28/90 days) mortality in patients with ACLF. Age, total bilirubin, international normalized ratio, and hepatic encephalopathy were independent risk factors for short- and long-term mortality risk in ACLF patients (*p* < 0.05).

**Conclusions:** Patients with DC-ACLF have a higher mortality rate, especially long-term mortality, compared to non-DC-ACLF patients. Therefore, DC should be included in the diagnostic criteria of APASL-ACLF and treated according to the ACLF management process.

## Introduction

Acute-on-chronic liver failure (ACLF) is an acute deterioration of chronic liver disease (CLD) with high short-term mortality (1). The current widely accepted diagnostic criteria for ACLF is derived from the Asia-Pacific Association for the Study of the Liver (APASL) (2) and the European Association for the Study of the Liver (EASL) (3), which have different definitions of chronic liver disease in addition to differences in the indicators of organ dysfunction or failure. APASL-ACLF includes non-cirrhotic chronic liver disease but not decompensated cirrhosis as representing “chronic” whereas EASL-ACLF includes only cirrhosis, either compensated or decompensated to define the chronic liver disease.

Since the etiology of chronic liver disease in the Chronic Liver Failure (CLIF) ACLF in Cirrhosis (CANONIC) (3) study defining EASL-ACLF was mostly non Hepatitis B virus (HBV) infection, Chinese scholars (4) conducted a prospective multicenter study in which they proposed that patients with high short-term mortality in the non-cirrhotic HBV population should be diagnosed with ACLF in addition to developed diagnostic criteria for HBV-related ACLF. To further improve the definition of ACLF, the World Gastroenterology Organization (WGO) (5) suggests that ACLF may be divided into 3 categories: type-A for patients without cirrhosis, type-B for well-compensated cirrhosis, and type-C for previous decompensated cirrhosis (DC). The application value of WGO type in HBV-related ACLF patients diagnosed by EASL-ACLF diagnostic criteria has been preliminarily verified, which can distinguish the precipitating events, organ failure, and short-term prognosis (6). However, whether DC (type C) should be included in the APASL-ACLF diagnostic criteria has not been conclusive. It was proposed in the consensus recommendations of the APASL for ACLF that CLD should not include decompensated cirrhosis, but the evidence grade for this recommendation is C (Low or very low quality), stating that the opinion requires confirmation by further studies (7).

With this large, retrospective, and multi-center cohort, we aimed to compare the clinical characteristics and prognosis of patients with DC and non-DC-ACLF defined by APASL, to provide an evidence-based basis for whether DC should be included in the diagnostic criteria of APASL-ACLF.

## Methods

### Study Design

We performed a retrospective cohort study using data from liver units in three university hospitals. Each liver unit had a regular ward, an intensive care unit, and a liver transplantation center. The same liver transplantation allocation policy was used at all study hospitals (8). Patients were screened from November 2012 to June 2019 in the Tianjin Third Central Hospital and the Fifth Medical Center of PLA General Hospital, and from January 2015 to June 2020 in Beijing You'an Hospital Affiliated with Capital Medical University. The study protocol was approved by the Clinical Research Ethics Committee of Beijing You'an Hospital Affiliated with Capital Medical University, the Tianjin Third Central Hospital, and the Fifth Medical Center of PLA General Hospital. Informed consent was waived due to the retrospective nature of this study.

### Patients

We screened patients hospitalized for at least 1 day with an acute hepatic insult (2) that occurs in patients with chronic liver disease (CLD) of all etiologies, manifested by jaundice [serum total bilirubin (TB) ≥ 5 mg/dl] and coagulation dysfunction [international normalized ratio (INR) ≥ 1.5], and complicated within 4 weeks by ascites and/or encephalopathy. The enrolment criteria were the fulfillment of the above indications, and the CLD included non-cirrhotic chronic liver disease, well-compensated cirrhosis, and previous decompensated cirrhosis (5). Cirrhosis was diagnosed based on previous liver biopsy results, or findings provided by laboratory test results, endoscopy, and radiologic imaging. The previous decompensation of cirrhosis was defined by the acute development of large ascites, hepatic encephalopathy, gastrointestinal hemorrhage, bacterial infection, or any combination of these, and presented at 6 months before the present episode.

The exclusion criteria were as follows: liver cancer or other malignant tumors, severe chronic extra-hepatic disease, pregnancy or lactation, and coinfection with HIV. All patient data were retrieved from electronic medical records. All treatments, mainly including etiological and comprehensive treatment, that were performed comply with the guidelines for ACLF which is accredited by the Chinese Medical Association (9).

### Data Collection

We collected data from all enrolled patients on medical history, detailed demographics, laboratory measurements, radiology results, and precipitating events of ACLF at the time of enrollment. Potential precipitating events included hepatitis B viral infection (HBV) reactivation (10), active alcoholism (3) (more than 14 drinks per week in women and more than 21 drinks per week in men within the previous 3 months), bacterial infection, and drug-induced liver injury. Information on liver transplantation and death were also collected from all enrolled patients and transplant-free survival/mortality was estimated for all enrolled patients at 28-, 90-, 180-, 360-, 540-, and 720-day after enrollment or ACLF diagnosis.

### Procedures

The APASL-ACLF criteria (2), with the exception of the diagnostic indicator of chronic liver disease, were used to assess and identify two groups: patients with ACLF with decompensated cirrhosis (DC-ACLF) and patients with ACLF with non-cirrhotic chronic liver disease or compensated cirrhosis (non-DC-ACLF). The clinical and laboratory characteristics of patients with ACLF were compared among the above two groups. The 28/90/180/360/540/720-day mortality (patients who received a liver transplant were considered dead) was profiled in two groups. Then, multivariate Cox PH regression analysis, including decompensation cirrhosis (DC) and other clinical measurements as candidate risk factors, was conducted to select the factors associated with short-term (28/90-day) and long-term (180/360/540/720-day) mortality.

### Statistical Analysis

Continuous variables were presented as Mean ± SD and median [interquartile range (IQR)], and categorical variables as *n* (%). Wilcoxon rank-sum and chi-squared tests were performed for continuous and categorical variables, respectively. Kaplan-Meier survival curves were plotted and compared with log-rank tests. Multivariate Cox proportional hazards (PH) models were fitted with a stepwise method using significant baseline factors (candidate variables included DC, complications, and laboratory measurements, *p* < 0.05) that had been pre-filtered in univariate PH models to identify the independent relationship between DC and mortality of patients with ACLF. Transplant patients were included in the mortality analysis. A two-sided *p* < 0.05 was considered statistically significant. All statistical analyses were performed with R × 64 4.0.3 (http://www.r-project.org/).

## Results

### Patients

A total of 689 patients were enrolled in the study. Table 1 shows the characteristics of the enrollment of the whole group. In total, 407 patients (59.1%) had an HBV etiology with a diagnosed history of CHB with the most frequent complications was bacterial infection (83.9%), followed by ascites (72.3%), hepatic encephalopathy (19.2%), and gastrointestinal hemorrhage (8.3%). Of the patients assessed using APASL-ACLF criteria without the diagnostic indicator of chronic liver disease, 435 (63.1%) patients without decompensation cirrhosis were diagnosed with ACLF (non-DC-ACLF), and 254 (36.9%) had the previous decompensation of cirrhosis (DC-ACLF). Overall, the 28-, 90-, 180-, 360-, 540-, and 720-day transplant-free survival rate in this study was 78.8, 61.3, 55.7, 48.9, 42.7, and 38.2%, respectively.

**Table 1:** Baseline characteristics of the study patients.

**Variables**	***N* = 689**
Age (y), median (IQR)	49 (41–57)
Male sex, *n* (%)	545 (79.1)
**Underlying chronic liver disease**, ***n*** **(%)**	
Compensated cirrhosis and non-cirrhosis	435 (63.1)
Decompensated cirrhosis	254 (36.9)
**Etiology of liver disease**, ***n*** **(%)**	
HBV	407 (59.1)
HCV	10 (1.5)
Alcohol	136 (19.7)
HBV + Alcohol	63 (9.1)
AIH	33 (4.8)
Others	40 (5.8)
**Precipitating events**, ***n*** **(%)**	
HBV reactivation	70 (10.1)
Bacterial infection	89 (12.9)
Alcohol	11 (1.6)
Drug	31 (4.5)
Unknown	488 (70.8)
**Laboratory data at admission, median (IQR)**	
ALT (U/L)	134 (47.2–440.0)
AST (U/L)	156.5 (85.2–398.8)
ALB (g/L)	28.9 (25.2–32.2)
TB (mg/dL)	16.4 (11.3–23.4)
INR	2.1 (1.8–2.6)
CR (mg/dL)	0.83 (0.64–1.04)
Na (mmol/L)	134.9 (131.2–137.6)
WBC (×10^9^/L)	6.8 (5.0–9.4)
PLT (×10^9^/L)	92 (62.5–132)
HGB (g/L)	119 (103–136)
**Scores at admission, median (IQR)**	
MELD score	23.1 (18.7–26.8)
MELD-Na score	25.4 (20.5–31.2)
**Complications**, ***n*** **(%)**	
Ascites	498 (72.3)
Bacterial infection	578 (83.9)
Gastrointestinal hemorrhage	57 (8.3)
Hepatic encephalopathy	132 (19.2)
**Transplant-free survival**, ***n*** **(%)**	
28-day	543 (78.8)
90-day	402 (61.3)
180-day	341 (55.7)
360-day	280 (48.9)
540-day	229 (42.7)
720-day	194 (38.2)

### Clinical Characteristics of ACLF Patients Stratified by Chronic Liver Disease

Demographics, laboratory data, scores (MELD scores and MELD-Na scores), and complications were compared between patients with non-DC-ACLF and DC-ACLF (Table 2). Compared with patients with non-DC-ACLF, those with DC-ACLF were more likely to be complicated with ascites (*p* < 0.05), gastrointestinal hemorrhage (*p* < 0.05). They had more severe disease as indicated by their significantly higher MELD scores [24.2 (IQR 19.5–27.2) vs. 22.7 (IQR 18.1–26.3)] and MELD-Na scores [26.9 (IQR 22.4–34.4) vs. 24.3 (IQR 20.0–29.5)] (both *p* < 0.05), and more severe kidney dysfunction as indicated by their significantly higher serum creatinine (mg/dl) [0.97 (IQR 0.73–1.15) vs. 0.76 (IQR 0.59–0.99)] and lower serum sodium [134 (IQR 129–137) vs. 135.3 (IQR 132.2–137.9)] (both *p* < 0.05). Coagulopathy (INR), liver (TB) dysfunction and precipitating events were not statistically different between the two groups (*p* > 0.05).

**Table 2:** Characteristics of acute-on-chronic liver failure (ACLF) patients with and without decompensated cirrhosis (DC).

**Variables**	**DC-ACLF (*N* = 254)**	**Non-DC-ACLF (*N* = 435)**	***P-*value**
Age (y), median (IQR)	49 (44–57)	48 (40–57)	0.065
Male sex, *n* (%)	206 (81.1)	339 (77.9)	0.323
**Precipitating events**, ***n*** **(%)**			0.203
Intrahepatic insults	35 (13.8)	79 (18.2)	
Extrahepatic insults	38 (15.0)	51 (11.7)	
Unknown	181 (71.3)	305 (70.1)	
**Laboratory data, median (IQR)**			
ALT (U/L)	111 (62–233.2)	176.7 (61.8–550.3)	0.000
AST (U/L)	58.5 (53.3–64.4)	186 (99.9–482)	0.000
ALB (g/L)	27.4 (23.6–30.8)	29.5 (25.7–32.7)	0.000
TB (mg/dL)	16.02 (10.4–22.9)	16.6 (11.6–23.7)	0.201
INR	2.14 (1.86–2.53)	2.17 (1.83–2.62)	0.686
CR (mg/dL)	0.97 (0.73–1.15)	0.76 (0.59–0.99)	0.000
Na (mmol/L)	134 (129–137)	135.3 (132.2–137.9)	0.000
WBC (×10^9^/L)	6.9 (5.1–9.9)	6.7 (4.9–8.9)	0.259
PLT (×10^9^/L)	73 (47.5–112.5)	103 (71.3–141.8)	0.000
HGB (g/L)	113 (95–131)	121 (106.1–139.0)	0.000
**Scores, median (IQR)**			
MELD score	24.2 (19.5–27.2)	22.7 (18.1–26.3)	0.032
MELD-Na score	26.9 (22.4–34.4)	24.3 (20.0–29.5)	0.000
**Complications**, ***n*** **(%)**			
Ascites	203 (79.9)	295 (67.8)	0.001
Bacterial infection	220 (86.6)	358 (82.3)	0.137
Gastrointestinal hemorrhage	30 (11.8)	27 (6.2)	0.010
Hepatic encephalopathy	55 (21.7)	77 (17.7)	0.203

*Intrahepatic insults include hepatitis B virus (HBV) reactivation, alcohol, hepatotoxic drugs*.

*Extrahepatic insults include bacterial infection*.

### Clinical Outcomes

**Figure 1:**
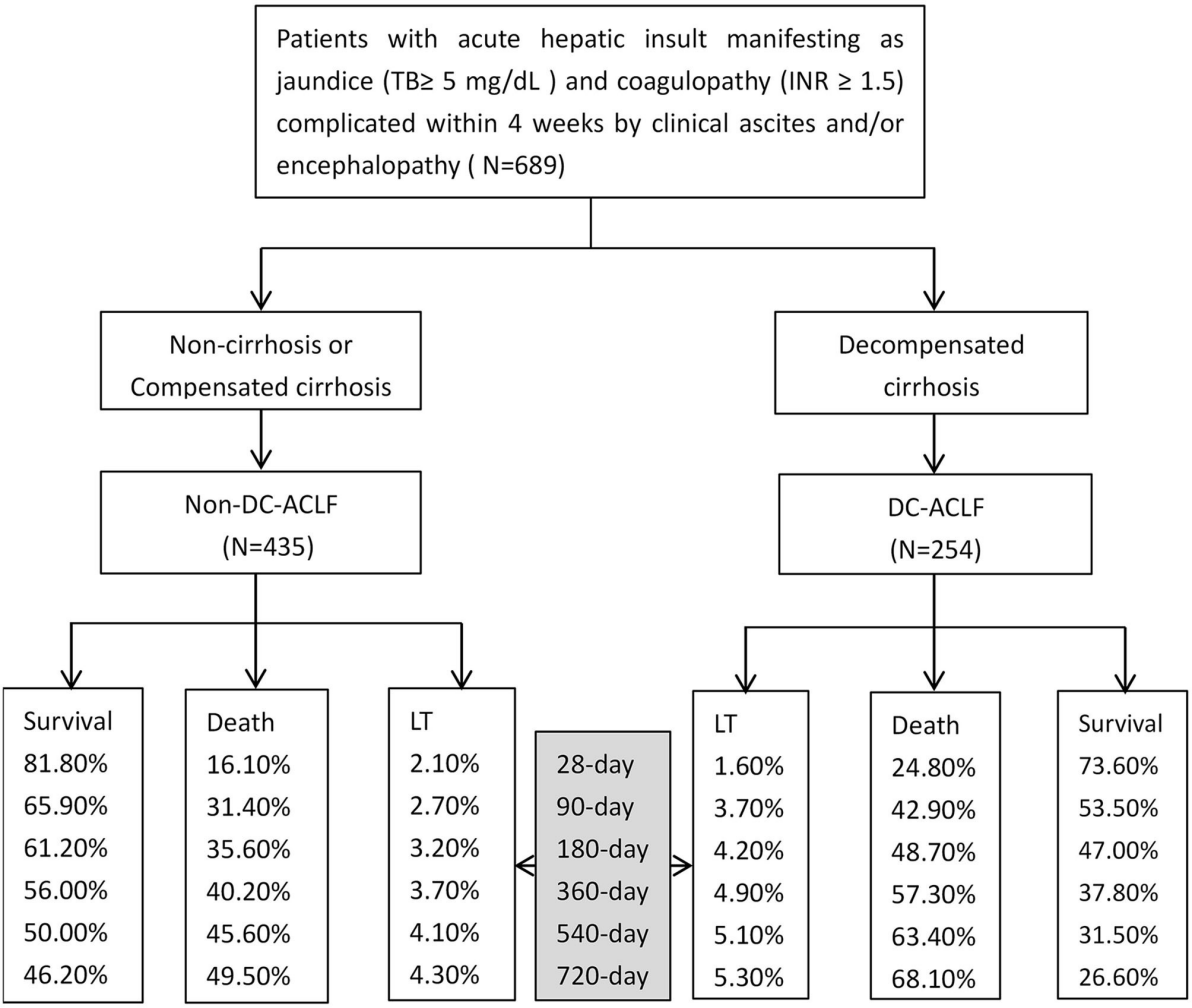
Acute-on-chronic liver failure (ACLF) with different underlying chronic liver diseases and associated outcomes since admission.

**Figure 2:**
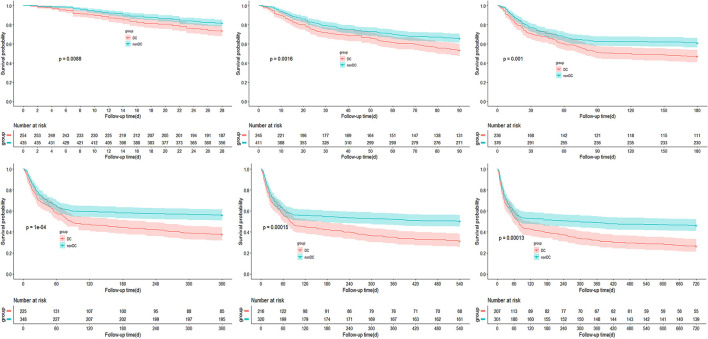
Incidence of transplant-free survival in ACLF patients with and without decompensated cirrhosis (DC).

In DC-ACLF group, 1.6, 3.7, 4.2, 4.9, 5.1, and 5.3% patients who received a liver transplant by 28-, 90-, 180-, 360-, 540-, and 720-day, respectively, whereas a total of 24.8, 42.9, 48.7, 57.3, 63.4, and 68.1% patients died without receiving a liver transplant for the same corresponding periods (Figure 1). The transplant-free survival was significantly lower in patients with DC-ACLF than in those without DC (non-DC-ACLF), irrespective of the diagnostic criteria (Figure 2), especially in terms of long-term transplant free survival, which was significantly different between the two groups with longer follow-up (720-day).

**Figure 3:**
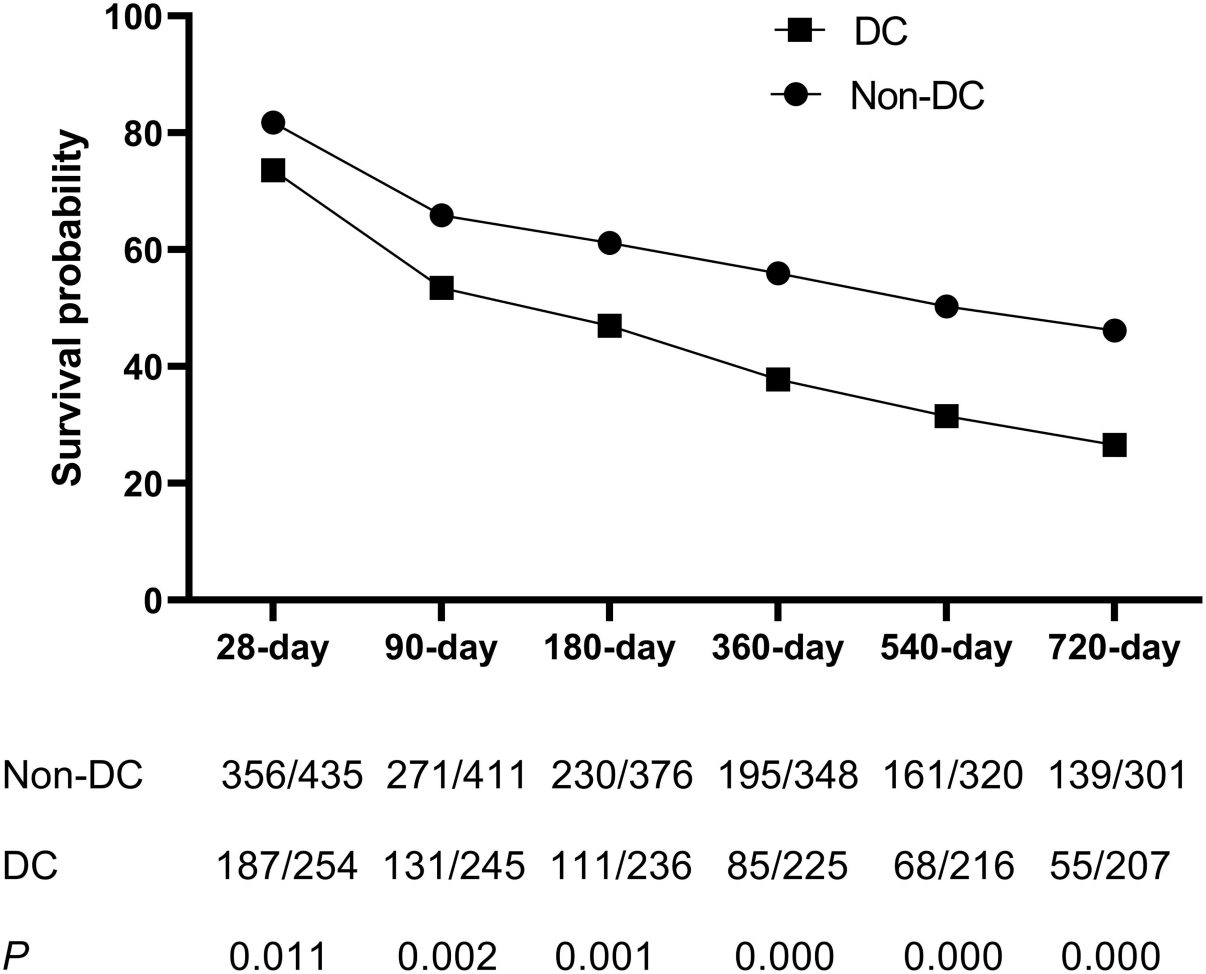
The survival analysis of patients with ACLF between DC group and non-DC group during 28, 90, 180, 360, 540, and 720 days.

Kaplan-Meier curves showing the transplant-free survival of ACLF patients with or without DC according to the APASL-ACLF are provided in Figure 3. At up to 720 days of follow-up, transplant free survival at all time periods (28, 90, 180, 360, 540, and 720 days) was significantly lower for patients in the DC group than for patients in the non-DC group (*p* < 0.05 by log-rank test).

### Decompensated Cirrhosis and Short- and Long-Term Mortality in Patients With ACLF

To clarify the independent relationship between DC and short and long-term outcomes in patients with ACLF, we performed univariate and multivariate Cox regression analyses of patients at various time periods of follow-up (Table 3). After adjusting for the factors that were statistically significant in the univariate regression analysis (*p* < 0.05), the results of multivariate analysis showed that patients without DC had a similar risk of death as those with DC on 28 days [aHR (95% CI) 0.717 (0.505–1.019), *p* > 0.05], and 90 days [aHR (95% CI) 0.753 (0.553–1.025), *p* > 0.05], implying that DC was not an independent risk factor for short-term mortality in ACLF patients.

**Table 3:** Adjusted HRs and 95% CI of decompensated cirrhosis (DC) for acute-on-chronic liver failure (ACLF) mortality by Cox proportional hazards model.

	**Univariate analysis**		**Multivariate analysis**	
**Non-DC vs. DC**	**HR (95% CI)**	***P-*value**	**aHR (95% CI)**	***P-*value**
28-day	0.649 (0.469–0.899)	0.009	0.717 (0.505–1.019)^a^	0.064
90-day	0.673 (0.525–0.862)	0.002	0.753 (0.553–1.025)^b^	0.052
180-day	0.672 (0.530–0.854)	0.001	0.712 (0.524–0.967)^c^	0.029
360-day	0.638 (0.507–0.803)	0.000	0.686 (0.512–0.920)^c^	0.011
540-day	0.651 (0.520–0.814)	0.000	0.701 (0.526–0.935)^d^	0.016
720-day	0.651 (0.522–0.813)	0.000	0.694 (0.523–0.922)^d^	0.012

*The multivariate logistic regression model was fitted with a stepwise selection method using clinically and statistically baseline factors that had been screened in univariate analysis*.

a *Adjusted for age, TB, INR, Na, Cr, MELD, MELD-Na, Bacteria, and HE*.

b *Adjusted for age, TB, INR, Na, Cr, PLT, MELD, MELD-Na, Bacteria, GIB, HE, and ascites*.

c *Adjusted for age, TB, INR, Na, Cr, ALT, PLT, HGB, MELD, MELD-Na, Bacteria, GIB, HE, and ascites*.

d *Adjusted for age, TB, INR, Na, Cr, ALT, PLT, WBC, HGB, MELD, MELD-Na, Bacteria, HE, and ascites*.

However, at time periods beyond 180 days of follow-up, patients in the non-DC group had a lower risk of death compared with those in the DC group (*p* < 0.05), with an adjusted HR (95%CI) of 0.712 (0.524–0.967), 0.686 (0.512–0.920), 0.701 (0.526–0.935), and 0.694 (0.523–0.922) by 180, 360, 540, and 720 days of follow-up, respectively. From this, DC increases the long-term risk of death in ACLF patients.

The results of univariate and multivariate Cox regression analyses of factors associated with mortality risk in ACLF at each follow-up time are detailed in Supplementary Tables 1–6, and it is important to note that age, TB, INR, and hepatic encephalopathy were independent risk factors associated with both short and long-term mortality risk in patients with ACLF (*p* < 0.05).

We separately analyzed the independent risk factors for short-term outcome in DC-ACLF and non-DC-ACLF patients and found that age, TB, and INR were the common short-term mortality risk factors in both groups (Supplementary Table 7). In addition, hepatic encephalopathy was a risk factor for short-term mortality in DC-ACLF patients.

## Discussion

When patients were diagnosed with ACLF according to EASL, the definition of CLD should include non-cirrhotic, compensated cirrhosis, and decompensated cirrhosis, an idea that has been confirmed in patients with HBV-related ACLF (4). Nevertheless, according to APASL, patients with a history of decompensated cirrhosis cannot be diagnosed with ACLF (2). In this retrospective study based on the enrolment standards of the APASL consortium definition of ACLF, most patients had HBV-related chronic liver disease (59.1%). When the APASL-ACLF criteria without non-DC were used, 245 additional patients with DC were diagnosed with ACLF. Baseline characteristics and short and long-term mortality were observed in populations with DC-ACLF. Compared with patients with non-DC-ACLF (fulfilling the APASL-ACLF criteria), those with DC-ACLF were more likely to be complicated with ascites, gastrointestinal hemorrhage, and had the more severe disease as indicated by their significantly higher MELD scores and MELD-Na scores, and more severe kidney dysfunction. The transplant free mortality rates of patients with DC-ACLF at 28, 90, 180, 360, 540, and 720 days were 24.8, 42.9, 48.7, 57.3, 63.4, and 68.1%, respectively, which were significantly higher than those of patients with non-DC-ACLF. Obviously, the 28 day mortality rate for patients with DC-ACLF is higher than the 15% used to develop the diagnostic criteria for ACLF (3, 4), and their 90 day mortality rate is higher than that of acute decompensation (23–29%) reported by APASL (2), indicating that patients with high short-term mortality in DC populations should be diagnosed with ACLF. The APASL guidelines emphasize that ACLF is a reversible syndrome, classifying acute liver injury occurring in patients with decompensated cirrhosis as chronic liver failure (CLIF), which also implies entry on the liver transplant waiting list. But it is noteworthy that the 1-year survival rate of patients with DC-ACLF in this study was 37.8% and this subset of patients is potentially reversible and does not urgently require liver transplantation. Therefore, managing DC-ACLF patients according to the ACLF management process facilitates organ allocation. Furthermore, the inclusion of DC in the APASL-ACLF definition expanded the application of ACLF definition (11) and was consistent with the WGO consensus (5).

Previous studies (12, 13) suggest that patients with previous DC had higher 90-day mortality than those without previous DC, and the prognostic model including DC showed excellent predictive value for 90-day mortality. This result was quite different from the results of the CANONIC study (3), in which the 90-day mortality of ACLF was higher in patients without previous acute decompensation (AD) than in patients with previous DC. Moreau (14) proposed that this can be explained by an inappropriate inflammatory response and a lack of tolerance to inflammation in patients without previous AD. The results presented in this study show that DC is not an independent risk factor for short-term mortality (28/90 days) but rather, a risk factor affecting the long-term outcome of patients (beyond 180 days). The controversial impact of decompensated cirrhosis on short-term outcomes may be explained by the different diagnostic criteria and etiologies of ACLF in different studies, but the results of DC on long-term mortality in ACLF are consistent. Some scholars (15, 16) prospectively followed up the long-term outcomes of ACLF patients and found that a prior history of AD is the most important factor affecting long-term mortality following an ACLF episode regardless of Model for End-stage Liver Disease score, considering that decreased hepatic reserve would be the predominant factor over inappropriate inflammatory response or ACLF severity with regard to the long-term outcome of patients who have survived ACLF.

In addition, our study found that age, TB, INR, and hepatic encephalopathy were independent risk factors associated with both short and long-term mortality in patients with ACLF. Among them, total bilirubin, INR, and hepatic encephalopathy were included in the APASL ACLF Research Consortium (AARC) score (17) used to manage APASL-ACLF, confirming again the prognostic importance of the above indicators.

This retrospective study with insufficient information does have its limitations in the nature of the study design. First, we did not observe changes in the dynamic clinical indicators of the patients, and it is possible that some potential influencing factors were ignored when analyzing the influencing factors on the long-term prognosis of ACLF patients. Second, a bacterial infection is judged according to the use of antibiotics, which is often related to the diagnosis and treatment experience of clinicians, leading to an overestimation of the bacterial infection rate in our patients. Finally, indeterminate precipitating events in our study denoted the absence of all previously described precipitating events. Since this study is a multicenter retrospective study, it is difficult to determine the precipitating events, especially as one of the centers did not give information about predisposing factors, which is also another limitation of our study. But this is a deletion from a single center and for the overall study population, it is not considered to be subject to deletion bias.

Future prospective studies of multicenter design are needed to verify the necessity of including DC in the diagnostic criteria of APASL. Additionally, it has been shown (18) that submassive hepatic necrosis could differentiate ACLF from AD. Thus, it is expected that DC-ACLF may be clarified by liver pathology in future studies.

## Conclusions

In conclusion, our study shows that patients with DC-ACLF have a higher mortality rate, especially long-term mortality, compared to non-DC-ACLF patients. Therefore, DC should be included in the diagnostic criteria of APASL-ACLF and treated according to the ACLF management process, which is clinically helpful for early diagnosis, management, and prognosis.

## Data Availability Statement

The original contributions presented in the study are included in the article/Supplementary Materials, further inquiries can be directed to the corresponding author/s.

## Ethics Statement

The studies involving human participants were reviewed and approved by Clinical Research Ethics Committee of Beijing You'an Hospital Affiliated to Capital Medical University. The Ethics Committee waived the requirement of written informed consent for participation.

## Author Contributions

MX and MK contributed to the conception and design of the study, drafted the initial manuscript, and reviewed and revised the manuscript. MX, MK, PY, FL, and YCa performed the initial data analysis and interpreted the data. SY, YZ, HZ, BZ, and WL coordinated and supervised the data collection and management. ZD and SX revised the manuscript critically for important intellectual content. YCh and TH contributed to the conception and design of the study and reviewed and revised the manuscript critically. All authors have read and approved the final manuscript.

## Funding

This work was supported by the National 13th 5-Year Plan for Hepatitis Research (2017ZX10203201-005 and 2017ZX10203201-007), National Key R&D Program of China (2017YFA0103000), Beijing Municipal Administration of Hospitals Clinical Medicine Development of Special Funding Support (ZYLX201806), and the National Natural Science Foundation of China (81870429).

## Conflict of Interest

The authors declare that the research was conducted in the absence of any commercial or financial relationships that could be construed as a potential conflict of interest.

## Publisher's Note

All claims expressed in this article are solely those of the authors and do not necessarily represent those of their affiliated organizations, or those of the publisher, the editors and the reviewers. Any product that may be evaluated in this article, or claim that may be made by its manufacturer, is not guaranteed or endorsed by the publisher.
