# Supplementary material for: Acute-On-Chronic Liver Failure Defined by Asian Pacific Association for the Study of the Liver Should Include Decompensated Cirrhosis
Source: Front Med (Lausanne). 2021 Oct 15;8:750061. doi: 10.3389/fmed.2021.750061 (PMC8554226; doi:10.3389/fmed.2021.750061)
Supplement: Supplementary file 1 [file Data_Sheet_1.docx]

Supplementary table 1 Univariate and multivariate Cox regression analysis for 28-day mortality in ACLF patients with and without DC

|  | Univariate analysis | | Multivariate analysis | |
| --- | --- | --- | --- | --- |
| Variables | HR (95% CI) | *P value* | aHR (95% CI) | *P value* |
| Age (y) | 1.030(1.010-1.040) | 0.000 | 1.033(1.018- 1.049) | 0.000 |
| Male | 1.280(0.881-1.870) | 0.194 |  |  |
| **non-DC vs. DC** | **0.649(0.469-0.899)** | **0.009** | **0.717(0.505-1.019)** | **0.064** |
| Laboratory data at admission |  |  |  |  |
| ALT (U/L) | 1.000(0.999-1.000) | 0.466 |  |  |
| AST (U/L) | 1.000(0.999-1.000) | 0.992 |  |  |
| ALB (g/L) | 1.000(0.963-1.040) | 0.980 |  |  |
| TB (mg/dL) | 1.050(1.030-1.060) | 0.000 | 1.041(1.022-1.061) | 0.000 |
| INR | 1.590(1.380-1.840) | 0.000 | 1.298(1.055-1.598) | 0.013 |
| CR (mg/dL) | 1.450(1.140-1.850) | 0.003 |  |  |
| Na (mmol/L) | 0.946(0.921-0.973) | 0.000 |  |  |
| WBC (×10^9/L) | 1.020(0.993-1.040) | 0.161 |  |  |
| PLT (×10^9/L) | 0.997(0.994-1.000) | 0.098 |  |  |
| HGB(g/L) | 0.993(0.987-1.000) | 0.059 |  |  |
| Scores at admission |  |  |  |  |
| MELD score | 1.120(1.080-1.150) | 0.000 | 1.057(1.024-1.092) | 0.000 |
| MELD-Na score | 1.060(1.050-1.080) | 0.000 |  |  |
| Complications |  |  |  |  |
| Ascites | 1.230(0.779-1.960) | 0.370 |  |  |
| Bacterial infection | 1.930(1.110-3.340) | 0.020 |  |  |
| Gastrointestinal hemorrhage | 1.490(0.885-2.500) | 0.134 |  |  |
| Hepatic encephalopathy | 2.880(2.050-4.040) | 0.000 | 2.013(1.385-2.927) | 0.000 |

Supplementary table 2 Univariate and multivariate Cox regression analysis for 90-day mortality in ACLF patients with and without DC

|  | Univariate analysis | | Multivariate analysis | |
| --- | --- | --- | --- | --- |
| Variables | HR (95% CI) | *P value* | aHR (95% CI) | *P value* |
| Age (y) | 1.030(1.020-1.040) | 0.000 | 1.031(1.017-1.044) | 0.000 |
| Male | 1.100(0.817-1.480) | 0.532 |  |  |
| **non-DC vs. DC** | **0.673(0.525-0.862)** | **0.002** | **0.753(0.553-1.025)** | **0.052** |
| Laboratory data at admission |  |  |  |  |
| ALT (U/L) | 1.000(0.999-1.000） | 0.105 |  |  |
| AST (U/L) | 1.000(0.999-1.000） | 0.354 |  |  |
| ALB (g/L) | 0.983(0.956-1.010) | 0.255 |  |  |
| TB (mg/dL) | 1.040(1.030-1.060) | 0.000 | 1.045(1.029-1.061) | 0.000 |
| INR | 1.350(1.190-1.540) | 0.000 | 1.279(1.037-1.578) | 0.022 |
| CR (mg/dL) | 1.420(1.160-1.730) | 0.001 |  |  |
| Na (mmol/L) | 0.952(0.932-0.972) | 0.000 |  |  |
| WBC (×10^9/L) | 1.010(0.985-1.030) | 0.600 |  |  |
| PLT (×10^9/L) | 0.997(0.995-1.000) | 0.042 |  |  |
| HGB(g/L) | 0.995(0.990-1.000) | 0.074 |  |  |
| Scores at admission |  |  |  |  |
| MELD score | 1.090(1.070-1.110) | 0.000 | 1.047(1.019-1.076) | 0.000 |
| MELD-Na score | 1.050(1.040-1.060) | 0.000 |  |  |
| Complications |  |  |  |  |
| Ascites | 1.630(1.120-2.380) | 0.010 |  |  |
| Bacterial infection | 1.710(1.160-2.540) | 0.007 |  |  |
| Gastrointestinal hemorrhage | 1.720(1.180-2.520) | 0.005 |  |  |
| Hepatic encephalopathy | 2.140(1.630-2.810) | 0.000 | 1.437 (1.385-2.927) | 0.041 |

Supplementary table 3 Univariate and multivariate Cox regression analysis for 180-day mortality in ACLF patients with and without DC

|  | | Univariate analysis |  | Multivariate analysis | |
| --- | --- | --- | --- | --- | --- |
| Variables | | HR (95% CI) | P value | aHR (95% CI) | P value |
| Age (y) | | 1.020(1.010-1.030) | 0.000 | 1.029(1.016-1.042) | 0.000 |
| Male | | 1.050(0.781-1.400) | 0.761 |  |  |
| **non-DC vs. DC** | | **0.672(0.530-0.854)** | **0.001** | **0.712(0.524-0.967)** | **0.029** |
| Laboratory data at admission | |  |  |  |  |
| ALT (U/L) | 1.000(0.999-1.000) | 0.045 |  |  | |
| AST (U/L) | 1.000(0.999-1.000) | 0.147 |  |  | |
| ALB (g/L) | 0.983(0.956-1.010) | 0.221 |  |  | |
| TB (mg/dL) | 1.040(1.030-1.050) | 0.000 | 1.040(1.024-1.057) | 0.000 | |
| INR | 1.340(1.180-1.510) | 0.000 | 1.257(1.031-1.533) | 0.024 | |
| CR (mg/dL) | 1.390(1.150-1.680) | 0.001 |  |  | |
| Na (mmol/L) | 0.944(0.925-0.964) | 0.000 |  |  | |
| WBC (×10^9/L) | 1.010(0.992-1.030) | 0.273 |  |  | |
| PLT (×10^9/L) | 0.998(0.996-1.000) | 0.107 |  |  | |
| HGB(g/L) | 0.994(0.989-0.999) | 0.013 |  |  | |
| Scores at admission | |  |  |  |  |
| MELD score | 1.090(1.060-1.110) | 0.000 | 1.049(1.022-1.079) | 0.000 | |
| MELD-Na score | 1.050(1.040-1.070) | 0.000 |  |  | |
| Complications | |  |  |  |  |
| Ascites | | 1.700(1.180-2.460) | 0.005 |  |  |
| Bacterial infection | | 1.990(1.350-2.950) | 0.001 | 1.769(1.037-3.017) | 0.036 |
| Gastrointestinal hemorrhage | | 1.570(1.080-2.280) | 0.018 |  |  |
| Hepatic encephalopathy | | 2.290(1.760-2.980) | 0.000 | 1.506(1.069-2.121) | 0.019 |

Supplementary table 4 Univariate and multivariate Cox regression analysis for 360-day mortality in ACLF patients with and without DC

|  | Univariate analysis | | Multivariate analysis | |
| --- | --- | --- | --- | --- |
| Variables | HR (95% CI) | *P value* | aHR (95% CI) | *P value* |
| Age (y) | 1.030(1.020-1.040) | 0.000 | 1.031(1.018-1.044) | 0.000 |
| Male | 1.020(0.770-1.360) | 0.876 |  |  |
| **non-DC vs. DC** | **0.638(0.507-0.803)** | **0.000** | **0.686(0.512-0.919)** | **0.011** |
| Laboratory data at admission |  |  |  |  |
| ALT (U/L) | 1.000(0.999-1.000) | 0.027 |  |  |
| AST (U/L) | 1.000(0.999-1.000) | 0.144 |  |  |
| ALB (g/L) | 0.974(0.947-1.000) | 0.053 |  |  |
| TB (mg/dL) | 1.040(1.030-1.060) | 0.000 | 1.047(1.029-1.066) | 0.000 |
| INR | 1.310(1.170-1.480) | 0.000 | 1.278(1.052-1.554) | 0.013 |
| CR (mg/dL) | 1.410(1.170-1.680) | 0.000 |  |  |
| Na (mmol/L) | 0.945(0.926-0.964) | 0.000 |  |  |
| WBC (×10^9/L) | 1.010(0.995-1.030) | 0.164 |  |  |
| PLT (×10^9/L) | 0.999(0.996-1.000) | 0.194 |  |  |
| HGB(g/L) | 0.993(0.988-0.998) | 0.005 |  |  |
| Scores at admission |  |  |  |  |
| MELD score | 1.080(1.060-1.100) | 0.000 | 1.031(1.003-1.060) | 0.032 |
| MELD-Na score | 1.050(1.040-1.060) | 0.000 |  |  |
| Complications |  |  |  |  |
| Ascites | 1.780(1.240-2.540) | 0.002 |  |  |
| Bacterial infection | 1.860(1.290-2.680) | 0.001 |  |  |
| Gastrointestinal hemorrhage | 1.510(1.050-2.190) | 0.027 |  |  |
| Hepatic encephalopathy | 2.300(1.780-2.970) | 0.000 | 1.727(1.247-2.391) | 0.001 |

Supplementary table 5 Univariate and multivariate Cox regression analysis for 540-day mortality in ACLF patients with and without DC

|  | Univariate analysis | | Multivariate analysis | |
| --- | --- | --- | --- | --- |
| Variables | HR (95% CI) | *P value* | aHR (95% CI) | *P value* |
| Age (y) | 1.030(1.020-1.040) | 0.000 | 1.031(1.019-1.043) | 0.000 |
| Male | 1.140(0.866-1.490) | 0.359 |  |  |
| **non-DC vs. DC** | **0.651(0.520-0.814)** | **0.000** | **0.704(0.526-0.935)** | **0.016** |
| Laboratory data at admission |  |  |  |  |
| ALT (U/L) | 1.000(0.999-1.000) | 0.011 |  |  |
| AST (U/L) | 1.000(0.999-1.000) | 0.064 |  |  |
| ALB (g/L) | 0.979(0.954-1.010) | 0.116 |  |  |
| TB (mg/dL) | 1.040(1.030-1.050) | 0.000 | 1.054(1.038-1.071) | 0.000 |
| INR | 1.270(1.130-1.430) | 0.000 | 1.382(1.179-1.619) | 0.000 |
| CR (mg/dL) | 1.330(1.110-1.600) | 0.002 |  |  |
| Na (mmol/L) | 0.948(0.929-0.967) | 0.000 |  |  |
| WBC (×10^9/L) | 1.020(1.000-1.040) | 0.044 |  |  |
| PLT (×10^9/L) | 0.999(0.997-1.000) | 0.236 |  |  |
| HGB(g/L) | 0.992(0.987-0.996) | 0.000 |  |  |
| Scores at admission |  |  |  |  |
| MELD score | 1.060(1.040-1.080) | 0.000 |  |  |
| MELD-Na score | 1.040(1.030-1.060) | 0.000 |  |  |
| Complications |  |  |  |  |
| Ascites | 1.700(1.200-2.400) | 0.003 |  |  |
| Bacterial infection | 1.980(1.370-2.860) | 0.000 | 1.713(1.032-2.844) | 0.037 |
| Gastrointestinal hemorrhage | 1.400(0.978-2.020) | 0.066 |  |  |
| Hepatic encephalopathy | 2.230(1.740-2.870) | 0.000 | 1.711(1.246-2.350) | 0.000 |

Supplementary table 6 Univariate and multivariate Cox regression analysis for 720-day mortality in ACLF patients with and without DC

|  | Univariate analysis | | Multivariate analysis | |
| --- | --- | --- | --- | --- |
| Variables | HR (95% CI) | *P value* | aHR (95% CI) | *P value* |
| Age (y) | 1.030(1.020-1.040) | 0.000 | 1.033(1.021-1.045) | 0.000 |
| Male | 1.180(0.903-1.540) | 0.226 |  |  |
| **non-DC vs. DC** | **0.651(0.522-0.813)** | **0.000** | **0.694(0.523-0.922)** | **0.012** |
| Laboratory data at admission |  |  |  |  |
| ALT (U/L) | 1.000(0.999-1.000) | 0.031 |  |  |
| AST (U/L) | 1.000(0.999-1.000) | 0.228 |  |  |
| ALB (g/L) | 0.978(0.953-1.000) | 0.083 |  |  |
| TB (mg/dL) | 1.040(1.020-1.050) | 0.000 | 1.043(1.027-1.061) | 0.000 |
| INR | 1.350(1.210-1.510) | 0.000 | 1.405(1.189-1.659) | 0.000 |
| CR (mg/dL) | 1.290(1.080-1.550) | 0.006 |  |  |
| Na (mmol/L) | 0.944(0.926-0.963) | 0.000 |  |  |
| WBC (×10^9/L) | 1.020(1.000-1.050) | 0.028 |  |  |
| PLT (×10^9/L) | 0.999(0.997-1.000) | 0.375 |  |  |
| HGB(g/L) | 0.992(0.988-0.997) | 0.002 |  |  |
| Scores at admission |  |  |  |  |
| MELD score | 1.070(1.040-1.090) | 0.000 |  |  |
| MELD-Na score | 1.050(1.030-1.060) | 0.000 |  |  |
| Complications |  |  |  |  |
| Ascites | 1.520(1.080-2.130) | 0.016 |  |  |
| Bacterial infection | 1.980(1.380-2.860) | 0.000 |  |  |
| Gastrointestinal hemorrhage | 1.360(0.951-1.960) | 0.092 |  |  |
| Hepatic encephalopathy | 2.130(1.660-2.740) | 0.000 | 1.529(1.116-2.098) | 0.008 |

Supplementary table 7. Multivariate analysis of independent risk factors of 28-day mortality in ACLF patients with or without DC by Cox proportional hazard model.

| Variables | aHR(95%CI) | P-value |
| --- | --- | --- |
| **ACLF without DC** |  |  |
| Age (y) | 1.0351.0118-1.059） | 0.003 |
| TB (mg/dL) | 1.051(1.0245-1.079) | 0.000 |
| INR | 1.743(1.2549-2.422) | 0.000 |
|  |  |  |
| **ACLF with DC** |  |  |
| Age (y) | 1.033(1.004-1.061) | 0.022 |
| TB (mg/dL) | 1.054(1.023-1.085) | 0.000 |
| INR | 1.622(1.235-2.131) | 0.001 |
| Hepatic encephalopathy | 1.897(1.055-3.412) | 0.032 |

For ACLF patients without DC, variables entering multivariate analysis were age, TB, INR, Na, Cr, MELD, MELD-Na, Bacteria and HE. For patients with DC, variables entering multivariate analysis were age, sex, TB, INR, MELD, MELD-Na and HE.
